# Supplementary material for: Content-rich biological network constructed by mining PubMed abstracts
Source: BMC Bioinformatics. 2004 Oct 8;5:147. doi: 10.1186/1471-2105-5-147 (PMC528731; doi:10.1186/1471-2105-5-147)
Supplement: Additional File 5 — The original Chilibot query results of the term "long-term potentiation (LTP)" and 22 other terms, limiting the latest references analyzed to the years 1990, 1995, 2000, and 2004. [file 1471-2105-5-147-S5.bz2 › chilibotAdditionalFile5/ltp2004/html/ERK_KV4.2.html]

 


 **ERK** and **KV4.2** 
  
Found 3 abstracts in PubMed,  **3 abstracts were retrieved and analyzed**.  


---

 Search Google  |
 PDF files only 
|  EDU domain only 

---

**Interactive relationship** (e.g. stimulation, inhibition, etc)

- The A type potassium channel  **Kv4.2**  is a substrate for the mitogen activated protein kinase  **ERK** .  Ref: 11080179 J Neurochem, 2000
- The sequence of  **Kv4.2**  contains several consensus sites for  **ERK**  phosphorylation.  Ref: 11080179 J Neurochem, 2000
- We have developed antibodies recognizing  **Kv4.2**  triply phosphorylated at the three  **ERK**  sites as well as two antibodies recognizing singly phosphorylated  **Kv4.2**  channels at the PKA sites one amino terminal and one carboxy terminal.  Ref: 11040264 Learn MemLearn Mem, 2000

**Parallel relationship** (e.g. studied together, co-existance, homology, etc.)

- We determined that this antibody recognizes  **ERK**  phosphorylated  **Kv4.2**  in COS 7 cells transfected with  **Kv4.2**  and native  **ERK**  phosphorylated  **Kv4.2**  in the rat hippocampus.  Ref: 11080179 J Neurochem, 2000
- Phosphorylation consensus sites for various kinases exist within the sequence of the potassium channel subunit  **Kv4.2** , including consensus sites for extracellular signal regulated kinase mitogen activated protein kinase  **ERK**  MAPK, protein kinase A PKA, protein kinase C PKC, and calcium calmodulin dependent kinase II CaMKII, and kinase assays have shown that particular amino acids of the consensus sites are bonafide phosphorylation sites in vitro.  Ref: 11040264 Learn MemLearn Mem, 2000
- In the present studies, we tested the hypothesis that  **Kv4.2**  is an  **ERK**  substrate.  Ref: 11080179 J Neurochem, 2000
- For example, the stratum lacunosum moleculare, which receives inputs from the entorhinal cortex via the perforant pathway, displays relatively little  **ERK**  phosphorylated  **Kv4.2**  or PKA carboxy terminal phosphorylated  **Kv4.2** .  Ref: 11040264 Learn MemLearn Mem, 2000
- Similarly, of the three antibodies tested, the soma of CA3 neurons are primarily recognized by the  **ERK**  triply phosphorylated  **Kv4.2**  antibody, and the mossy fiber inputs to CA3 are primarily recognized by the carboxy terminal PKA phosphorylated  **Kv4.2** .  Ref: 11040264 Learn MemLearn Mem, 2000
- These observations indicate that  **Kv4.2**  is a substrate for  **ERK**  in vitro and in vivo, and suggest that  **ERK**  may regulate potassium channel function by direct phosphorylation of the pore forming alpha subunit.  Ref: 11080179 J Neurochem, 2000
